# Mastering cardiomyocyte mitophagy: molecular governance, pathological derailment and therapeutics

**DOI:** 10.7717/peerj.20700

**Published:** 2026-02-10

**Authors:** Pan Liu, Haosheng Wu, Huanhuan Ren, Jing Wang, Fan Yang

**Affiliations:** 1 Zhongyi School, Anhui University of Chinese Medicine, Hefei, China; 2 Great Health Research, Center for Xin’an Medicine and Modernization of Traditional Chinese Medicine of IHM, Xian, China; 3 Department of Cardiology, The First Affiliated Hospital of Anhui University of Chinese Medicine, Hefei, China

**Keywords:** Mitophagy, Cardiomyocyte, Cardiac hypertrophy and heart failure, Cardiac aging

## Abstract

Mitophagy is a pivotal quality control pathway that maintains cardiac energy metabolism and structural stability by selectively removing damaged or senescent mitochondria, thereby keeping mitochondrial dynamics in balance. This process secures cardiomyocyte survival, calcium handling, and contractile function during both rest and stress. When mitophagic flux is inadequate, accumulation of reactive oxygen species, disruption of calcium homeostasis, and uncontrolled inflammation act together to drive pathological hypertrophy, heart failure, cardiac aging, and obesity-associated cardiomyopathy. Conversely, appropriate activation of mitophagy can lessen structural injury and restore pump performance during ischemia reperfusion, pressure overload, and metabolic stress. This review summarizes the central regulatory network of cardiac mitophagy and its pathological roles across cardiovascular disorders, emphasizing that careful modulation of flux is essential for preserving myocardial homeostasis. Recent experimental strategies that target mitophagy are also discussed, providing a theoretical foundation for the development of precise cardioprotective therapies.

## Survey methodology

PubMed database was used for related literature search using the keywords “cardiomyocyte”, “mitophagy”, “heart disease”, “cardiovascular disease”.

## Introduction

Mitochondria are central to cardiac bioenergetics, supplying most of the adenosine triphosphate (ATP) that sustains continuous contraction. Beyond energy production, they regulate key cellular pathways, including the generation of metabolic intermediates, detoxification of harmful byproducts, and initiation of apoptosis (Bravo-San Pedro, Kroemer & Galluzzi, 2017). Even modest disturbances in mitochondrial function can impair cardiomyocyte performance and viability. Dysfunctional mitochondria not only limit ATP synthesis but also heighten oxidative stress (Bhatti, Bhatti & Reddy, 2017; Chan, 2006). Because mitochondria are tightly coupled to the endoplasmic reticulum (ER), mitochondrial defects also perturb Ca^2+^ homeostasis; the shift from physiological to pathological Ca^2+^ signaling is now recognized as a disease hallmark (Berridge, 2016).

Maintaining mitochondrial quality therefore requires stringent intracellular control. Damaged organelles are removed by mitophagy, a process directed by nuclear-encoded proteins (Li et al., 2021). Canonical routes comprise the PINK1/Parkin axis and several receptor-mediated pathways; emerging work also implicates Beclin 1, cardiolipin (CL), and lipid droplet-associated processes in fine-tuning mitophagy. As a selective form of autophagy, mitophagy preserves cellular homeostasis by clearing injured or senescent mitochondria (Hamacher-Brady & Brady, 2016). When this surveillance falters, defective mitochondria accumulate, promoting oxidative stress, inflammation, and cardiomyocyte death (Palikaras, Lionaki & Tavernarakis, 2018; Xu, Shen & Ran, 2020). Robust mitophagy is indispensable for cardiovascular homeostasis and cardioprotection, and its impairment recurs in cardiac hypertrophy and failure, cardiac aging, and obesity-associated cardiomyopathy (Morales et al., 2020).

Although prior reviews have summarized cardiac mitophagy, most emphasize the canonical PINK1/Parkin cascade or hypoxia-linked receptors and provide limited integration across disease stages or guidance for translation. For example, Morales et al. (2020) outline mechanisms and broad disease links yet do not consolidate non-canonical modules such as Beclin 1/PI3K/ULK1 signaling, cardiolipin externalization, and crosstalk between lipid droplets and mitochondria within a cardiac framework. This review integrates canonical and non-canonical pathways, aligns mitophagy phenotypes with hypertrophy and heart failure, aging, and metabolic cardiomyopathy, and proposes a translational roadmap linking mechanism to practice. We first outline the molecular circuitry that governs mitophagy, then examine how its imbalance precipitates pathological remodeling, and finally synthesize current evidence that connects mitophagy dysregulation to prevalent heart diseases. We conclude by summarizing emerging therapeutic strategies that target key mitophagy regulators, with the aim of accelerating clinical translation of mitophagy-centered interventions for cardiovascular disorders. This review addresses mitochondrial and cardiovascular researchers and clinicians who seek to apply recent insights to patient care.

## Cellular defenses against mitochondrial injury: core mitophagy pathways

When mitochondrial lesions overwhelm cellular repair capacity, they pose a serious threat to cell integrity. Under these conditions, terminally damaged organelles are uncoupled from the functional mitochondrial network, sequestered within vesicles bounded by double membranes, and delivered to lysosomes for degradation through a process known as mitophagy.

### PINK1 and Parkin dependent mitophagy

**Figure 1:**
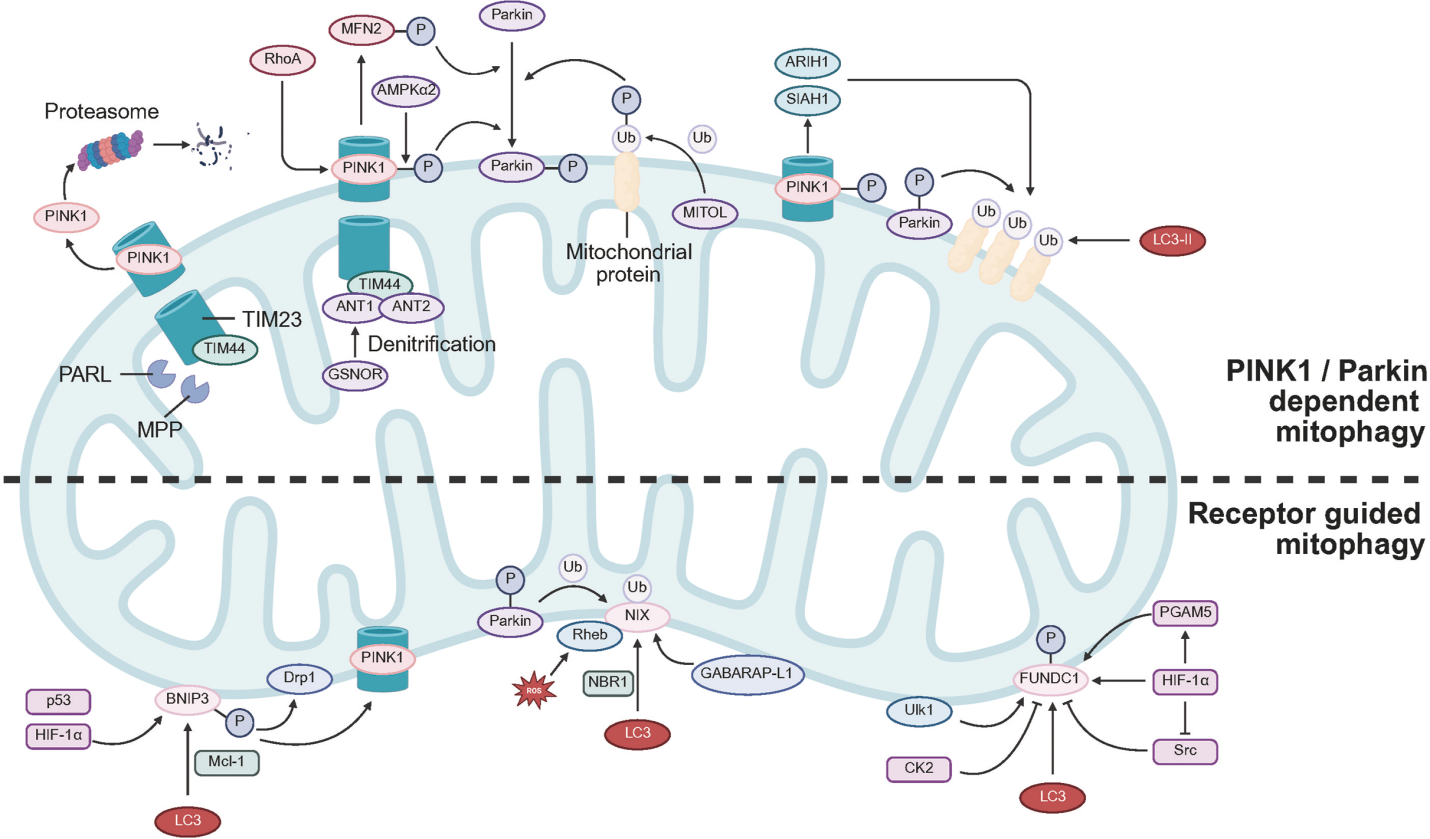
PINK1–Parkin dependent and receptor-mediated mitophagy pathways. PINK1/Parkin dependent mitophagy: In healthy mitochondria, PINK1 is imported and cleaved by PARL and MPP, released from the mitochondrial membrane, and degraded in the cytosol by the ubiquitin–proteasome system. Loss of mitochondrial membrane potential causes ANT1/ANT2–Tim23–Tim44 complexes to retain PINK1 on the OMM and block its import into the inner membrane. GSNOR-mediated denitrosylation of ANT1 and the small GTPase RhoA further stabilize PINK1 on the surface of cardiac mitochondria. On the OMM, PINK1 associates with TOM, undergoes autophosphorylation, and can be additionally phosphorylated by AMPKα2, while PINK1-dependent phosphorylation of MFN2 promotes Parkin recruitment. The OMM E3 ubiquitin ligase MITOL provides basal ubiquitination of PINK1-pathway components, thereby facilitating PINK1 phosphorylation. Accumulation of phosphorylated ubiquitin and PINK1 then recruits and activates cytosolic Parkin *via* phosphorylation of its ubiquitin-like domain, leading to the build-up of active PINK1 and Parkin on the mitochondrial surface. Parkin ubiquitinates multiple OMM proteins, and the E3 ligases ARIH1 and SIAH1 are subsequently recruited in a PINK1-dependent manner to further ubiquitinate mitochondrial substrates and fully engage mitophagy. Receptor guided mitophagy: BNIP3, transcriptionally induced by HIF-1α and p53, drives mitophagy under hypoxic stress. BNIP3 promotes Drp1 translocation to mitochondria, thereby coupling mitochondrial fission with autophagy in cardiomyocytes. By interacting with PINK1, BNIP3 enhances PINK1 accumulation on the OMM and facilitates Parkin recruitment. Under energetic stress and mitochondrial damage, Mcl-1 binds BNIP3 and, *via* its LIR motif, acts as an adaptor to reinforce cardiac mitophagy. BNIP3L/NIX mediates programmed mitochondrial clearance by recruiting GABARAP-L1 and, together with ROS-driven co-recruitment of Rheb and LC3, promotes mitophagosome formation. NIX further engages the PINK1–Parkin pathway: Parkin ubiquitinates NIX, enabling recruitment of the selective autophagy receptor NBR1, which bridges ubiquitin and LC3/GABARAP to assemble autophagosomes around mitochondria, and NIX also promotes Parkin targeting to depolarized mitochondria. The OMM receptor FUNDC1 mediates hypoxia-induced mitophagy through phosphorylation-dependent regulation: under normoxia, CK2 and Src phosphorylate FUNDC1 and weaken its binding to LC3, whereas under hypoxia reduced Src activity and dephosphorylation of FUNDC1 by PGAM5 stabilize FUNDC1–LC3 interactions. This process is antagonized by BCL2L1/Bcl-xL, which disrupts the FUNDC1–PGAM5 interaction under normoxic conditions. Hypoxia additionally recruits ULK1 to fragmented mitochondria, where it phosphorylates FUNDC1 to further increase LC3 affinity and promotes autophagosome formation and mitochondrial clearance. PARL, presenilin-associated rhomboid-like protease; MPP, mitochondrial processing peptidase; ANT1/ANT2, adenine nucleotide translocase 1/2; Tim23, translocase of inner mitochondrial membrane 23; Tim44, translocase of inner mitochondrial membrane 44; OMM, outer mitochondrial membrane; GSNOR, S-nitrosoglutathione reductase; TOM, translocase of the outer mitochondrial membrane complex; MITOL, mitochondrial ubiquitin ligase (MARCH5); ARIH1, ariadne RBR E3 ubiquitin protein ligase 1; SIAH1, seven *in absentia* homolog 1; Mcl-1, myeloid cell leukemia 1; LIR, LC3-interacting region; BNIP3L, BCL2/adenovirus E1B 19 kDa-interacting protein 3-like; NIX, NIP3-like protein X; GABARAP-L1, gamma-aminobutyric acid receptor-associated protein-like 1; Rheb, Ras homolog enriched in brain; NBR1, next to BRCA1 gene 1 protein; GABARAP, gamma-aminobutyric acid receptor-associated protein; FUNDC1, FUN14 domain containing 1; CK2, casein kinase 2; PGAM5, phosphoglycerate mutase family member 5; BCL2L1, B-cell lymphoma 2-like protein 1; ULK1, Unc-51-like autophagy activating kinase 1.

The PINK1 and Parkin dependent pathway of mitophagy has been characterized in detail. In mitochondria that remain functionally intact, nascent PINK1 undergoes sequential proteolysis (Fig. 1). It is first cleaved within the inner membrane by the presenilin associated rhomboid like protease (PARL) and subsequently within the matrix by mitochondrial processing peptidase (MPP) (Jin et al., 2010). The truncated form of PINK1 detaches from the membrane and is removed in the cytosol through the ubiquitin proteasome system (Narendra et al., 2010). When the mitochondrial membrane potential dissipates, import of PINK1 ceases and the protein accumulates on the outer mitochondrial membrane (OMM) (Narendra et al., 2010). This retention is facilitated by adenine nucleotide translocases ANT1 and ANT2, which associate with Tim23 and Tim44 at the inner membrane to block PINK1 translocation into the intermembrane space and thereby stabilize the kinase at the organelle surface (Hoshino et al., 2019). Detritylation of ANT1 at Cys160 by mitochondrial S-nitrosoglutathione reductase further supports this stabilization. Downregulation of S-nitrosoglutathione reductase in hypertrophic cardiomyocytes intensifies mitochondrial dysfunction and diminishes autophagy, underscoring the role of ANT1 in promoting mitophagy (Tang et al., 2023). In addition, activation of the small GTPase RhoA can anchor PINK1 on cardiac mitochondria independently of membrane depolarization, recruit Parkin, and enhance mitophagic flux (Tu et al., 2022). At the OMM, PINK1 binds to the translocase of the outer membrane complex (Lazarou et al., 2012), where it becomes catalytically competent through autophosphorylation (Eldeeb et al., 2024). The active kinase then phosphorylates preexisting ubiquitin at Ser65 on surface proteins of the organelle (Koyano et al., 2014). AMPKα2 can also phosphorylate PINK1 at Ser495 and promote cardiomyocyte mitophagy (Wang et al., 2018a). Furthermore, PINK1 dependent phosphorylation of MFN2 facilitates Parkin recruitment to mitochondria, enabling perinatal clearance of fetal cardiac mitochondria and establishing conditions for metabolic maturation (Gong et al., 2015). Basal ubiquitination of these proteins by the resident OMM E3 ligase MITOL is thought to prime them for subsequent PINK1 phosphorylation and the onset of mitophagy (Koyano et al., 2019). Phosphorylated ubiquitin serves as a signal that directs cytosolic Parkin to damaged mitochondria (Kane et al., 2014). PINK1 also phosphorylates the ubiquitin like domain of Parkin at Ser65, which further activates the ligase (Kondapalli et al., 2012). Active PINK1 and Parkin thus accumulate on the mitochondrial surface, generating an autocatalytic phosphor-ubiquitin amplification loop (Ordureau et al., 2014). Parkin conjugates ubiquitin to several outer membrane proteins, notably the fusion regulators MFN1 and MFN2, the trafficking adaptor MIRO, and the voltage dependent anion channel VDAC (Poole et al., 2010). The cytosolic E3 ligases ARIH1 and SIAH1 are likewise recruited to damaged mitochondria in a PINK1 dependent manner and add further ubiquitin tags to outer membrane substrates, thereby stimulating mitophagy (Szargel et al., 2016). Deubiquitinates such as USP8, USP15, USP30, and USP35 modulate this process by removing nonspecific ubiquitin modifications and sharpening autophagic selectivity (Cornelissen et al., 2014; Durcan et al., 2014; Gersch et al., 2017; Wang et al., 2015). However, they do not recognize phosphorylated ubiquitin or polyubiquitin chains, a limitation that preserves and amplifies the mitophagic signal (Harper, Ordureau & Heo, 2018).

Accumulating evidence indicates that PINK1 and Parkin play pivotal roles in directing mitochondrial degradation in response to developmental cues and cellular stress. Extensive studies in cardiomyocytes show that mitophagy driven by the PINK1 and Parkin pathway is elicited by mitochondrial dysfunction and by diverse pathological stresses (Hoshino et al., 2013; McWilliams et al., 2018). Because cardiac mitochondria are precisely organized to ensure rapid ATP distribution, defective organelles must detach from this ordered network and move to locations that favor degradation. Rho GTPase activating protein 26 is a direct PINK1 substrate. Phosphorylation of Rho GTPase activating protein 26 by PINK1 induces local F actin depolymerization, which facilitates the release of damaged mitochondria (Zhu et al., 2023). Substantial mechanistic insight has arisen from experiments using CCCP, a protonophore that abolishes the mitochondrial membrane potential Δψm and activates the integrated stress response (Koncha et al., 2021). Nevertheless, the extent to which the PINK1 and Parkin pathway operates under more physiological forms of stress remains unclear. *In vivo* studies in Drosophila and mice have questioned its role in maintaining mitochondrial quality (Lee et al., 2018) and in limiting mtDNA mutation accumulation under basal conditions (Filograna et al., 2024). Moreover, PINK1 appears dispensable for mitophagy in highly oxidative tissues (Kubli et al., 2015), which suggests that this pathway functions predominantly when mitochondrial injury is overt.

Although PINK1 is essential for canonical Parkin mediated mitophagy, Parkin can also translocate to mitochondria through alternative mechanisms. The 70 kDa heat shock protein 1L enhances Parkin recruitment, whereas the cochaperone BAG4 inhibits it (Hasson et al., 2013). HSP72 moves to mitochondria after skeletal muscle injury and facilitates Parkin docking (Drew et al., 2014), and the HSP70 cochaperone BIS co-translocates with Parkin to cardiac mitochondria to execute mitophagy (Tahrir et al., 2017). Conversely, Bcl-xL, Mcl-1, and p53 can block Parkin translocation (Yu et al., 2020a). Beyond direct regulation of Parkin recruitment, other mitophagy pathways may operate in parallel or under distinct stress conditions. For instance, tumor necrosis factor receptor associated factor 2 (TRAF2) can localize to mitochondria independently of Parkin to promote cardiomyocyte mitophagy (Yang et al., 2015). Hearts from Traf2 deficient mice display swollen mitochondria with sparse cristae and impaired mitophagy (Ma et al., 2022).

Notwithstanding extensive delineation of the PINK1 and Parkin pathway in depolarization induced mitophagy, its robustness and generalizability under physiological conditions remain debated. For example, loss of membrane potential after CCCP treatment, while invaluable for mapping the molecular cascade, represents a strong stress paradigm that may not faithfully capture cardiomyocyte responses under physiological or sublethal pathological settings. Consistent with this view, several *in vivo* studies have questioned the contribution of this pathway to steady state mitochondrial quality control. In Drosophila and mouse models, loss of PINK1 and Parkin function did not markedly compromise basal mitochondrial function or morphology (Lee et al., 2018), and its capacity to constrain mtDNA mutation burden appeared limited (Filograna et al., 2024). In addition, in highly oxidative tissues, PINK1 deficiency did not substantially impede cardiomyocyte mitophagy (Kubli et al., 2015). Taken together, these observations suggest that the PINK1 and Parkin route is preferentially engaged under conditions of overt mitochondrial injury, acting more as a module that responds to stress than as a routine mechanism for basal homeostasis. Future work should define activation thresholds and regulatory features under physiological and sub-pathological contexts and clarify how the contribution of this pathway varies across tissues, developmental stages, and disease settings.

### Receptor guided ubiquitin independent mitophagy

Receptor mediated mitophagy constitutes a ubiquitin independent pathway for mitochondrial clearance. It relies on dedicated anchors in the outer mitochondrial membrane whose LC3 interacting regions bind LC3 on the phagophore, thereby driving autophagosome formation (Gustafsson & Dorn, 2019). Compared with the PINK1/Parkin axis, the regulatory logic of this route is less clearly defined. To date, BNIP3 (BCL 2/adenovirus E1B 19 kDa interacting protein 3), NIX (also called BNIP3 like), and FUNDC1 (FUN14 domain containing protein 1) are the best characterized receptors. BNIP3 and FUNDC1 are engaged mainly under hypoxic or related stress (Liu et al., 2012), whereas NIX is indispensable for developmentally programmed mitophagy (Lampert et al., 2019). Each receptor plays critical roles in cardiomyocyte mitophagy (Dorn, 2010; Xu et al., 2022; Zhou et al., 2017).

BNIP3. BNIP3 participates in mitochondrial turnover during hypoxia (Liu et al., 2019). Under such stress, BNIP3 expression increases and its C terminal transmembrane domain anchors the protein in the outer mitochondrial membrane, exposing an N terminal LC3 interacting region to the cytosol (Kubli et al., 2013). Phosphorylation of Ser17 and Ser24 adjacent to this region is required for LC3 engagement (Zhu et al., 2013). In mouse embryonic fibroblasts, hypoxia inducible factor 1α upregulates BNIP3 and thereby drives mitophagy during hypoxia (Zhang et al., 2019a). In the heart, p53 likewise elevates BNIP3, promoting mitochondrial dysfunction and autophagy dependent cell death, a major facet of p53 activity in cardiomyocytes (Wang et al., 2013).

BNIP3 also stimulates mitophagy through Drp1 mediated fission, because BNIP3 recruits Drp1 to mitochondria and couples fission with autophagic removal (Lee et al., 2011). Excessive activation of this pathway can be harmful, since silencing BNIP3 mitigates doxorubicin induced mitochondrial fragmentation and cell death (Dhingra et al., 2017). The polyphenol ellagic acid similarly downregulates BNIP3 and the associated maladaptive autophagy, reducing mitochondrial injury and necrotic loss in cardiomyocytes, a property that may be beneficial during chemotherapy or ischemic stress (Dhingra et al., 2017). BNIP3 physically associates with PINK1, which facilitates its retention on the outer mitochondrial membrane and subsequent Parkin recruitment (Zhang et al., 2016); nonetheless, BNIP3 driven mitophagy can proceed without Parkin. The anti-apoptotic Bcl 2 family member Mcl-1 interacts with BNIP3 under energetic stress to enhance cardiac mitophagy (Moyzis et al., 2022). Mcl-1 contains an LC3 interacting motif and, acting as a BNIP3 adaptor, suppresses basal autophagy through Beclin 1 while selectively modulating mitophagy. Post translational regulation also contributes: UBXD8 recruits the hexametric AAA ATPase VCP to mitochondria and, together with an E3 ligase, accelerates BNIP3 degradation, thereby adjusting mitophagic activity (Zheng et al., 2022b).

BNIP3/NIX. Sharing 53% to 56% homology with BNIP3, NIX governs programmed mitochondrial clearance during reticulocyte maturation and erythroid differentiation (Sandoval et al., 2008). In CCCP treated cells, NIX recruits GABARAP L1 to stimulate mitophagy (Novak et al., 2010). Phosphorylation of Ser34 and Ser35 near the LIR stabilizes the interaction between NIX and LC3 (Rogov et al., 2017). Like BNIP3, the C-terminal region forms homodimers that are essential for the initiation of mitophagy (Marinković, Šprung & Novak, 2021). Accumulated reactive oxygen species from oxidative phosphorylation trigger co-recruitment of Rheb, NIX, and LC3, which promotes mitophagosome formation (Melser et al., 2013). In cardiac progenitor cells, mitophagy directed by BNIP3 and NIX reorganizes the mitochondrial reticulum, supports accurate differentiation into cardiomyocytes, and improves survival within infarcted myocardium (Lampert et al., 2019). Evidence from several studies indicates that NIX integrates with the PINK1/Parkin pathway. Parkin attaches ubiquitin to NIX and recruits the selective autophagy receptor NBR1, which links the ubiquitin signal to LC3 or GABARAP and initiates assembly of mitophagosomes around impaired mitochondria (Gao et al., 2015). NIX also promotes Parkin docking on depolarized organelles (Ding et al., 2010). The SCF^FBXL4^ ubiquitin ligase complex governs the localization of BNIP3 and NIX on the mitochondrial surface; loss of FBXL4 unleashes excessive mitophagy and results in perinatal lethality (Cao et al., 2023). In addition, the outer membrane protein TMEM11 forms a complex with BNIP3 and NIX at sites of mitophagosome formation. TMEM11 deficiency under normoxic conditions or simulated hypoxia produces hyperactive mitophagy, which indicates that TMEM11 spatially restrains clearance driven by BNIP3 and NIX (Yamada et al., 2018).

FUNDC1. FUNDC1 is an outer mitochondrial membrane receptor whose N-terminal LIR binds LC3 directly and orchestrates hypoxia-induced mitophagy (Liu et al., 2012). Its activity is finely tuned by phosphorylation. Under normoxia, casein kinase 2 phosphorylates Ser13 and Src kinase phosphorylates Tyr18, which weakens LC3 binding (Chen et al., 2014; Liu et al., 2012). Hypoxia lowers Src activity and Tyr18 phosphorylation, thereby stabilizing the interaction between FUNDC1 and LC3 (Liu et al., 2012). At the same time, the phosphatase PGAM5 dephosphorylates Ser13 to augment mitophagy, an effect that is blocked in normoxia by BCL2L1/Bcl-xL, which prevents association of PGAM5 and FUNDC1 (Wu et al., 2014a). Upon hypoxia or loss of Δψm, Ulk1-mediated phosphorylation of FUNDC1 at Ser17 further increases LC3 affinity and facilitates autophagosome assembly (Wu et al., 2014b). Functionally, FUNDC1-mediated mitophagy promotes differentiation and survival of stem and progenitor cells after myocardial infarction (Lampert et al., 2019). Hypobaric-hypoxia preconditioning activates this pathway and alleviates post-infarction dysfunction and fibrosis in mice (Li et al., 2022b).

Receptor-mediated mitophagy represents a ubiquitin-independent pathway for mitochondrial clearance, primarily driven by OMM-anchored proteins such as BNIP3, NIX, and FUNDC1. These receptors engage autophagosome formation through their LC3-interacting regions, which directly bind to LC3 on the phagophore membrane. BNIP3 and NIX play essential roles under hypoxic stress and during erythroid differentiation, and emerging evidence suggests they may functionally intersect with the PINK1–Parkin pathway, indicating they are not entirely independent. FUNDC1 undergoes multilayered regulation *via* site-specific phosphorylation, enabling dynamic control of mitophagy, particularly under cardiac hypoxia where it exerts cardioprotective effects. However, it remains unclear whether these receptors operate in a consistent manner across different cell types, and the precise modes of coordination with the Parkin-dependent pathway are yet to be fully defined. Moreover, the *in vivo* functions of upstream regulators such as TMEM11 and UBXD8 have not been systematically characterized, limiting our comprehensive understanding of the regulatory network governing this pathway.

### Alternative routes to mitochondrial clearance

#### Beclin 1 driven mitophagy initiation

**Figure 2:**
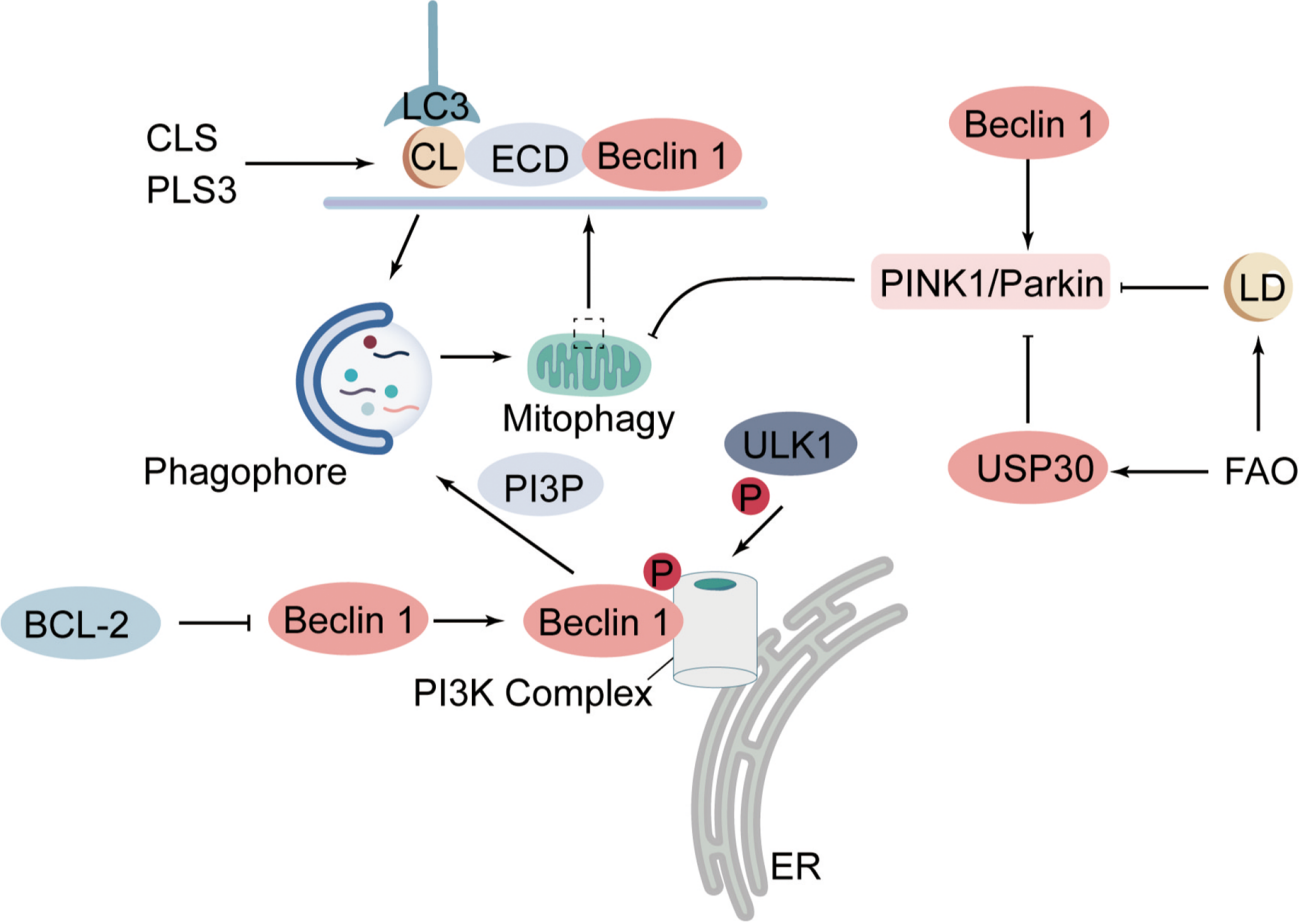
Roles of Beclin 1, cardiolipin and lipid droplets in mitophagy. Beclin 1 participates in assembling the class III PI3K complex, which is activated by phosphorylation through the serine-threonine kinase ULK1 to generate PI3P, thereby initiating autophagosome formation. Beclin 1 interacts with the apoptosis regulator BCL-2, whose sequestration of Beclin 1 suppresses its autophagy-initiating capacity. Beclin 1 also enhances PINK1–Parkin-mediated mitophagy, limiting the release of mitochondrial damage-associated molecular patterns. CL is a phospholipid normally located in the IMM. Upon OMM rupture, CL becomes exposed to the cytosol and, through binding to LC3, promotes autophagosome formation around damaged mitochondria. CLS and the phospholipid acyltransferase PLS3 further elevate CL levels on the OMM. CL can also interact with Beclin 1 and LC3 to aid autophagosome biogenesis, and Beclin 1 preferentially binds CL-rich membranes *via* its ECD. A high-fat diet drives LD accumulation and suppresses PINK1/Parkin-dependent mitophagy. FAO likewise inhibits mitophagy by allowing the deubiquitinase USP30 to remove ubiquitin chains and blockade the PINK1/Parkin pathway. PI3K, phosphatidylinositol 3-kinase; ULK1, Unc-51-like autophagy-activating kinase 1; PI3P, phosphatidylinositol-3-phosphate; BCL-2, B-cell lymphoma 2; PINK1, PTEN-induced kinase 1; Parkin, E3 ubiquitin-protein ligase Parkin; CL, cardiolipin; IMM, inner mitochondrial membrane; OMM, outer mitochondrial membrane; LC3, microtubule-associated protein 1A/1B-light chain 3; CLS, cardiolipin synthase; PLS3, phospholipid scramblase 3; ECD, evolutionarily conserved domain; LD, lipid droplet; FAO, fatty-acid oxidation; USP30, ubiquitin-specific protease 30.

Beclin 1 serves as the core scaffold of the class III PI3K complex that nucleates autophagosome formation. After phosphorylation by ULK1, the complex generates PI3P to recruit early autophagy factors, thereby initiating and extending the phagophore. During mitophagy, this process occurs adjacent to damaged mitochondria, positioning nascent autophagosomes for selective sequestration (Itakura et al., 2012; Jaber et al., 2012; Gelmetti et al., 2017; Quiles et al., 2023) (Fig. 2). BCL-2 binding to Beclin 1 restrains initiation, whereas weakening this interaction in a knock-in mouse elevates basal autophagy and extends lifespan, demonstrating *in vivo* that Beclin 1 availability gates flux (Fernández et al., 2018; Liang et al., 2001). In the heart, Beclin 1 is rapidly induced during pressure overload, ischemia-reperfusion, and myocardial infarction, in parallel with heightened autophagy and mitophagy (Matsui et al., 2007; Zhu et al., 2007; Maejima et al., 2013). Beclin 1 also augments PINK1/Parkin-dependent mitophagy in sepsis, limiting mitochondrial DAMP release and myocardial inflammation (Sun et al., 2018). Collectively, these findings identify the Beclin 1/PI3K initiation module as a central, druggable control point for tuning cardiac mitophagy.

#### Cardiolipin triggered mitophagy signaling

CL is a phospholipid normally confined to the mitochondrial inner membrane (IMM); only after injury-induced rupture of the OMM does CL become exposed to the cytosol, where its interaction with LC3 promotes the formation of autophagosomes around damaged mitochondria (Shen et al., 2017). LC3, the mammalian homolog of autophagy-related protein 8 (Atg8), is conjugated to phosphatidylethanolamine upon autophagy induction to generate lipidated LC3-II, a process essential for phagophore membrane extension and cargo recognition (Li et al., 2015). LC3 contains basic surface patches that bind CL with high specificity (Chu et al., 2013). Reducing CL biosynthesis, either by silencing cardiolipin synthase or the phospholipid acyl transferase PLS3, depletes CL on the OMM and markedly suppresses mitophagy (Chu et al., 2013). CL may also facilitate autophagosome biogenesis through coordinated interactions with Beclin 1 and LC3. Beclin 1 preferentially associates with CL-rich membranes through its evolutionarily conserved domain, where three aromatic residues create a hydrophobic patch that serves as the binding site (Huang et al., 2012). Because both the ER and mitochondria donate membrane fragments to the nascent phagophore, Beclin 1 could act as a molecular bridge, channeling mitochondrial membranes into the developing autophagic structure. The subsequent interaction between CL and LC3 further drives phagophore elongation, consistent with the central role of LC3 in membrane growth (Füllgrabe, Klionsky & Joseph, 2014). Autophagic precursors can nevertheless form without LC3 lipidation. *In vitro* studies using Atg5- or Atg3-deficient HeLa cells and mouse embryonic fibroblasts (MEFs) have demonstrated LC3-independent phagophore formation (Kishi-Itakura et al., 2014), which implies that complementary mechanisms support membrane expansion during autophagy (Li et al., 2015). Functionally, mesenchymal stem cells (MSCs) isolated from high-fat diet–induced obese mice (MSC^Ob^) contain less cardiolipin and exhibit a diminished capacity to clear damaged mitochondria compared with MSCs from chow-fed controls (Sagar et al., 2023).

#### Lipid droplet directed regulation of mitophagy

Obesity and diabetes are prevalent metabolic disorders that frequently lead to diastolic dysfunction, myocardial hypertrophy, and inflammation, changes collectively referred to as diabetic cardiomyopathy (Tan et al., 2020). In diabetic cardiomyopathy, insufficient insulin redirects myocardial substrate use from glucose toward fatty acids. The consequent reliance on fatty acid oxidation for ATP supply increases reactive oxygen species (ROS) formation and damages mitochondria (Kim, Wei & Sowers, 2008). These dysfunctional organelles can no longer balance fatty acid uptake with oxidation, resulting in lipid-droplet accumulation and lipotoxicity (D’Souza, Nzirorera & Kienesberger, 2016). Growing evidence indicates that LD-induced mitophagy is pivotal for maintaining cardiac mitochondrial integrity (Zhi et al., 2023). Tong et al. (2019) reported rapid LD deposition in the myocardium of mice fed a high-fat diet for 2 months; concurrently, active PINK1/Parkin-dependent mitophagy was progressively suppressed. Ablation of Atg7 or Parkin magnified LD overload and further impaired diastolic performance, whereas TAT-Beclin 1 re-initiated mitophagy, removed damaged mitochondria, and reduced LD content (Tong et al., 2019). In a TFEB^−/−^ heart model, Trivedi et al. (2020) observed prominent LD clustering with little change in mitophagy-related genes, suggesting that TFEB chiefly remodels lipid metabolism rather than directly modulating the canonical autophagy pathway to safeguard mitochondrial quality. Likewise, 24 weeks of high-fat feeding caused LD deposition, structural mitochondrial injury, and abnormal PINK1 expression, indicating that LD overload contributes to mitophagy imbalance (Zheng et al., 2023). Simvastatin substantially increased myocardial LD energy buffering while activating autophagy and mitophagy, easing Ang II-induced mitochondrial damage and improving cardiac function (Hsieh et al., 2019). Mechanistic work further shows that blockade of fatty-acid beta oxidation (FAO) alone can repress mitophagy. Sun et al. (2025) demonstrated that FAO deficiency in CPT2^−/−^ hearts interrupts the PINK1/Parkin pathway through USP30-mediated deubiquitination; inhibition of USP30 restores mitophagy, reduces LD burden, and markedly improves cardiac performance. In clinically relevant HFpEF models, heavy reliance on FAO blunts the mitophagy response and allows damaged mitochondria to accumulate, whereas enhancing FAO simultaneously normalizes mitophagy and improves diastolic function (Yoshii et al., 2024). Collectively, under lipid overload or defective lipid metabolism, timely activation of mitophagy that limits LD build-up and toxicity acts as a critical safeguard against metabolic cardiomyopathy. Targeting LD-reprogramming signals to re-establish synergy between LDs and mitophagy may provide a promising therapeutic avenue for metabolic heart failure.

In summary, beyond the classical receptor-mediated mechanisms, mitochondrial clearance can also be mediated by non-canonical signaling pathways involving Beclin 1, cardiolipin, and LDs. Beclin 1 coordinates autophagosome initiation through regulation of the PI3K complex, ensuring the selective sequestration of damaged mitochondria. Its interaction with BCL-2 modulates autophagic activity and plays a critical regulatory role in various cardiac disease settings. Cardiolipin, once externalized to the outer mitochondrial membrane following injury, directly interacts with LC3 and functions as a key signal for mitochondrial degradation. Its cooperation with Beclin 1 facilitates phagophore membrane elongation; however, the presence of LC3-independent mechanisms suggests that the regulatory network governing this process remains incompletely defined. Lipid droplets, which reflect underlying metabolic dysregulation, exhibit a reciprocal relationship with mitochondrial damage. LDs-induced mitophagy serves as an essential mechanism to alleviate lipotoxicity and maintain cardiac function. Although these alternative pathways are increasingly recognized as important modulators of mitochondrial quality control, their precise roles under different pathological conditions remain insufficiently elucidated. Further research is particularly needed to clarify the interplay between LDs signaling and the PINK1/Parkin pathway, as well as to determine whether Beclin 1 alone is sufficient to initiate mitochondria-specific autophagy.

## Pathogenic mechanisms of mitophagy imbalance

### Mitophagy in oxidative stress responses

**Figure 3:**
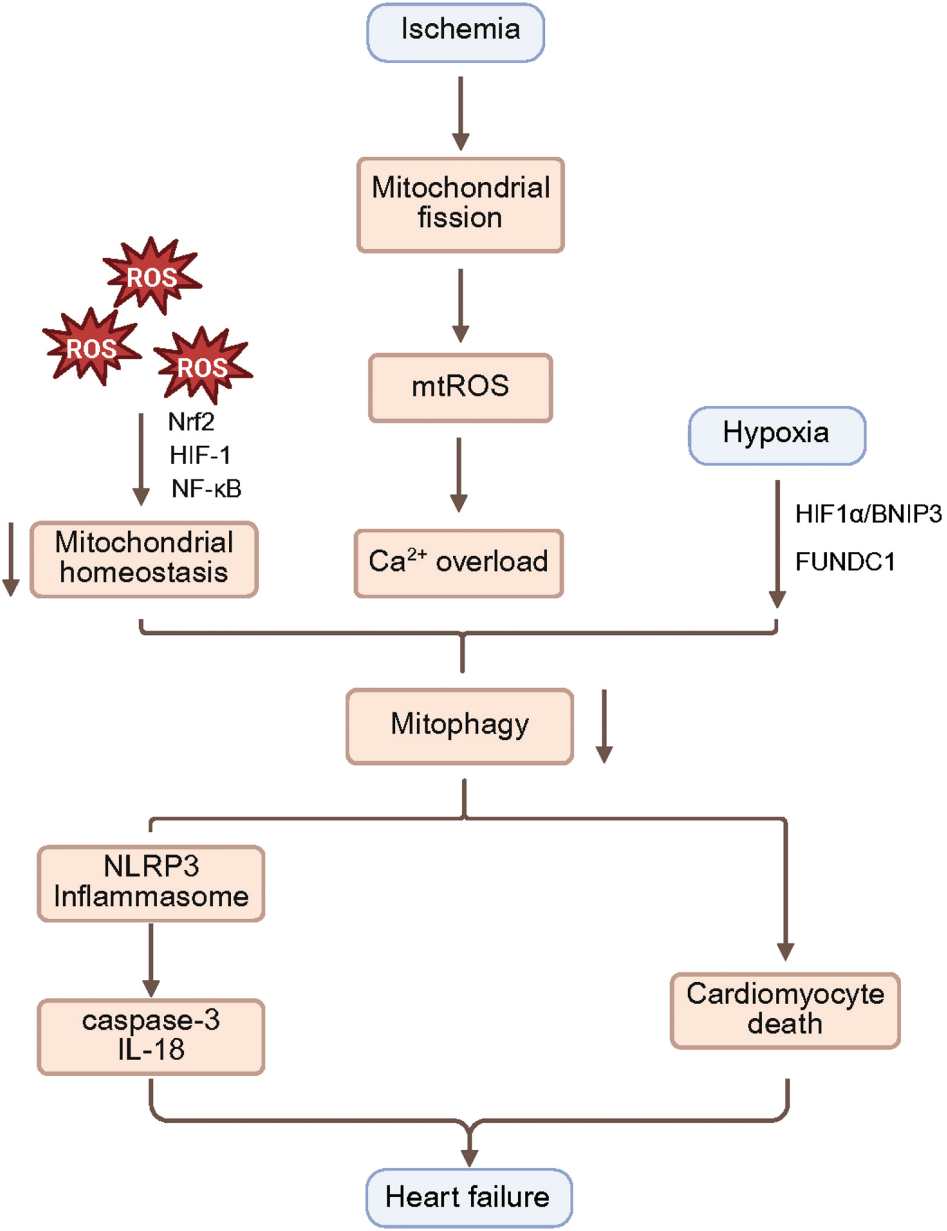
Pathological mechanisms linking mitophagy to oxidative stress, inflammation and cardiomyocyte death during I/R injury. I/R injury and acute myocardial infarction are accompanied by increased myocardial ROS, disruption of mitochondrial homeostasis, and activation of mitochondria-related signaling pathways including Nrf2, HIF-1 and NF-κB. During ischemia, enhanced mitochondrial fission leads to excessive production of mtROS, which promotes mitochondrial Ca²^+^ overload and triggers mitophagy. Hypoxia also engages mitophagy *via* the HIF-1α/BNIP3 axis and the mitochondrial receptor FUNDC1. Mitophagy, in turn, can drive activation and assembly of the NLRP3 inflammasome in cardiomyocytes, leading to upregulation of the pro-inflammatory mediators caspase-3 and IL-18. Appropriately activated PINK1/Parkin dependent mitophagy supports cardiomyocyte survival, attenuates cellular stress, apoptosis, inflammation and fibrosis, and thereby ameliorates heart failure. I/R, ischemia–reperfusion; mtROS, mitochondrial reactive oxygen species; BNIP3, BCL2/adenovirus E1B 19 kDa-interacting protein 3; FUNDC1, FUN14 domain containing 1; NLRP3, NOD-like receptor family pyrin domain containing 3; PINK1, PTEN-induced kinase 1.

Cardiomyocytes trigger an early stress response after injury, which elevates mtROS and impairs oxidative phosphorylation (Rabinovich-Nikitin et al., 2021). *In vivo*, models of ischemia-reperfusion, acute myocardial infarction, and heart failure consistently exhibit marked elevations in myocardial ROS (Peoples et al., 2019). *In vitro*, hypoxia, inflammation, or hyperglycemia increase ROS production and disrupt mitochondrial homeostasis (Fig. 3) (Shadel & Horvath, 2015). Even at low levels, ROS activate mitochondrial signaling mediated by Nrf2, HIF-1, and NF-κB (Donohoe et al., 2019). A recent study reported that the traditional Yiqi-Huoxue formula, a TCM regimen conceptually targeting qi augmentation and blood-flow promotion, alleviates hypoxia-induced myocardial injury and oxidative stress by modulating mitophagy (Chen et al., 2023), underscoring the central role of mitophagy in hypoxia-related cardiovascular damage. Moderate expression of mitochondria-targeted catalase suppresses ROS build-up, membrane depolarisation, and structural injury; when overexpressed it still lowers ROS but fails to restore mitochondrial function or prevent apoptosis, indicating that an appropriate ROS level is essential for mitochondrial equilibrium (Dong et al., 2022). Quercetin mitigates mitochondrial oxidative injury and limits cardiomyocyte necrosis after ischemia–reperfusion *via* the DNA-PKcs/SIRT5 axis (Chang et al., 2024), whereas DUSP12 attenuates ischemia–reperfusion damage by enhancing mitophagy (Cheng et al., 2023). Conversely, blocking mitophagy has been reported to decrease apoptosis and oxidative stress and to restore cardiac function (Bai et al., 2022). Suppression of Parkin-mediated mitophagy, however, causes excessive ROS accumulation, worsens myocardial injury, and accelerates heart failure (Jiang et al., 2022). Under hypoxia, autophagy driven by the HIF-1/BNIP3 pathway is tightly linked to reperfusion damage, and modulation of HIF-1α–BNIP3 lessens ischemia–reperfusion injury (Chen et al., 2024). In platelets, hypoxia-induced FUNDC1-dependent autophagy also diminishes reperfusion injury (Xu et al., 2024). In addition, Sirt6-enriched exosomes enhance mitophagy through the p62/Beclin-1 pathway and suppress AIM2-mediated pyroptosis, thereby improving ischemia–reperfusion outcomes (Liu et al., 2024). As a secondary calcium reservoir, mitochondria buffer cytosolic Ca^2+^ oscillations; excessive Ca^2+^ disrupts oxidative phosphorylation and ATP synthesis, compromising contractile performance (Bradshaw, Yoshida & Ling, 2012). During ischemia, mitochondrial fission increases, mitochondrial DNA becomes unevenly distributed among daughter organelles, transcription of respiratory chain proteins declines, and mtROS surges (Qin et al., 2013). mtROS oxidizes the mitochondrial Ca^2+^ uniporter, keeping it open and driving mitochondrial Ca^2+^ overload (Chen et al., 2022). Oxidation of the sarcoER Ca^2+^-ATPase at Cys674 further aggravates intracellular Ca^2+^ loading (Tong et al., 2008). Although mitophagy efficiently removes damaged mitochondria and lowers mtROS, direct evidence for its influence on calcium homeostasis remains limited. Moderate Ca^2+^ accumulation may stimulate mitochondrial biogenesis, while Nrf1/2 exert antioxidant effects by upregulating genes such as MnSOD, catalase, Prx3/5, UCP2, Trx2, and TrxR (Yeon et al., 2023). Zinc is also crucial in redox regulation: ZIP7 knockdown elevates mitochondrial Zn^2+^, induces depolarization, accumulates PINK1/Parkin, reduces ROS, and shrinks infarct size (Zhang et al., 2021). Excessive HIF-1 activation, Ca^2+^ overload, and autophagy dysregulation all exacerbate reperfusion damage, and iron metabolism plays a pivotal part in mitophagy-related ischemic injury of the heart (Corradi et al., 2023).

### Mitophagy and cardiac inflammatory signaling

In a neonatal cardiomyocyte model of simulated ischemia, mitophagy has been shown to trigger NLRP3 inflammasome activation (Wang et al., 2024). Mechanistically, Drp1-driven mitochondrial fission allows mtDNA and mtROS to escape into the cytosol, thereby fostering inflammasome assembly within cardiomyocytes and up-regulating pro-inflammatory mediators such as caspase 3 and interleukin-18 (Marek-Iannucci et al., 2019). Beyond cardiomyocytes, mitophagy markedly affects endothelial inflammation: under hypoxia, dysregulated autophagy exacerbates endothelial barrier injury and heightens monocyte–endothelial adhesion (Chu et al., 2024). Because mitophagy curtails the leakage of mtDNA and mtROS, its anti-inflammatory efficacy has been verified in several neurological disorders. Studies on PGC-1α- or Nrf2-dependent mitochondrial biogenesis and inflammation have so far centered on diabetic cardiomyopathy (Zheng, Chen & Sha, 2014). Recent work indicates that molecular hydrogen confers protection in an ischemic cardiomyopathy model by activating PINK1/Parkin-dependent mitophagy (Liu et al., 2023). The depolarizing agent CCCP can also reactivate the PINK1/Parkin pathway, counteracting the inhibition of mitophagy and mitochondrial function caused by Shenlian extract, a proprietary TCM compound preparation, whereas Shenlian extract may itself lessen the post-reperfusion no-reflow phenomenon by modulating mitophagy and dampen inflammation (Liu et al., 2023). Despite these advances, additional studies are needed to delineate precisely how mitophagy governs cardiac function and inflammatory signaling.

### Mitophagy regulated cardiomyocyte death

After acute myocardial infarction, TBC1D15 initiates lysosome-dependent mitophagy flux by regulating Fis1/RAB7-mediated contacts between mitochondria and lysosomes (Xu, Shen & Ran, 2020). Parkin simultaneously attaches ubiquitin to cyclophilin D in the necrotic signaling cascade, thereby preventing mPTP opening, limiting necrosis, and improving ventricular function (Ma et al., 2023). Up-regulation of the mitochondrial calcium uniporter (MCU) suppresses autophagy through the calpain/OPA1 route while relying on OPA1 to curb excessive apoptosis (Choi et al., 2021). Thyroid hormone enhances PINK1-dependent autophagy activity, lessens cell death, and confers cardioprotection (Yao et al., 2019). miR-494-3p lowers PGC-1α, modulates autophagy, and thus attenuates ischemia/reperfusion (I/R) injury while restraining apoptosis (Li et al., 2023). Hydrogen-rich saline activates the PINK1/Parkin pathway, reducing both inflammation and apoptosis (Yu et al., 2020b). Rhuscoriaria flavonoid RR protects the myocardium by inhibiting USP33, which promotes autophagy and suppresses apoptosis (Sun et al., 2019). During the early reperfusion period, activating G-protein-coupled estrogen receptor 1 (GPER1) decreases autophagy by down-regulating the PINK1/Parkin axis and protects cardiomyocytes, whereas pinocembrin acts through the same axis to augment autophagy and mitigate I/R injury (Guan et al., 2019). Although dysregulated Drp1-dependent mitochondrial fission interferes with autophagy, it paradoxically raises cellular tolerance to lethal hypoxia (Su et al., 2023). Most studies agree that moderate PINK1/Parkin-mediated mitophagy during pre-ischemic stress is pro-survival. RIPK3 up-regulation blocks this mitophagy by inhibiting AMPK, thereby fostering necroptosis and maladaptive remodeling; silencing RIPK3 prevents necrosis and eases hypoxic injury (Mu et al., 2023). Because myocardial infarction induces metabolic stress that activates AMPK, it is plausible that AMPK activation feeds back to reinforce PINK1/Parkin-directed mitophagy and delays cell death (Feng et al., 2017). Within this AMPK signaling axis, melatonin has been shown to stimulate the AMPK/OPA1 pathway, boost mitophagy, and promote cardiomyocyte survival, whereas loss of SIRT6 weakens these benefits (Zhu et al., 2021). Melatonin can also act through the Apelin/SIRT3 axis to restrain excessive autophagy and avert reperfusion damage (Enzan et al., 2023). Therapeutic hypothermia markedly amplifies mitophagy, reduces stress, apoptosis, inflammation, and fibrosis, highlighting cold-induced autophagy as a critical component of post-infarction repair and a potential strategy for preventing heart failure (Wang et al., 2022). During reperfusion, RIPK3 up-regulation phosphorylates FUNDC1, disrupts FUNDC1-mediated autophagy, and triggers apoptosis, whereas RIPK3 knock-down blocks this cascade and lessens cardiomyocyte loss (Zhang et al., 2019b).

Mitophagy imbalance contributes to the progression of cardiac injury through multiple mechanisms, encompassing oxidative stress, inflammatory signaling, and regulated cell death. Under oxidative stress, moderate activation of mitophagy facilitates the clearance of ROS, attenuates mitochondrial calcium overload and structural damage, and thereby stabilizes cardiomyocyte function. However, the direct relationship between mitophagy regulation and calcium homeostasis remains poorly defined, and both excessive and insufficient ROS levels may disrupt mitochondrial integrity. In the context of inflammation, mitophagy exerts anti-inflammatory effects by limiting the release of mtDNA and mtROS, thereby suppressing NLRP3 inflammasome activation. Nonetheless, the cell type–specific regulatory mechanisms of mitophagy in cardiomyocytes and endothelial cells require further clarification. Regarding cell death, appropriately regulated PINK1/Parkin-dependent mitophagy can inhibit both apoptosis and necrosis, enhancing myocardial resilience to stress. In contrast, aberrant activation of factors such as RIPK3 and Drp1 impairs mitophagy and aggravates cardiomyocyte loss. Currently, the functional outcomes of mitophagy under different pathological conditions remain inconsistent. The interplay between mitophagy and cell death transitions, as well as its integration with metabolic signals such as AMPK, warrants in-depth investigation to enable precise therapeutic modulation of mitophagy in cardiovascular disease.

## Mitophagy imbalance across cardiac diseases

### Mitophagy dynamics in hypertrophy and heart failure

Cardiac hypertrophy can be elicited by hemodynamic overload, neuro-humoral activation, ischemia, and other stressors. Initially, it is a compensatory adaptation to increased wall stress and oxygen demand, manifested by cardiomyocyte hypertrophy, structural remodeling, and increased total cardiac mass (Frey et al., 2004). Sustained pathological stimulation leads to maladaptive growth and apoptosis, ultimately progressing to heart failure (Burchfield, Xie & Hill, 2013). Throughout this process, mitochondrial energetics are crucial, as the hypertrophic myocardium requires substantially greater ATP, Ca^2+^ buffering, and ion transport to sustain pump performance (Huss & Kelly, 2005). Givvimani et al. (2012) improved left-ventricular function in the ascending-aortic ligation model by inhibiting DRP1 with mdivi-1 and observed parallel declines in LC3 and p62. By contrast, Ashrafian et al. (2010) showed that DRP1-mutant mice, with fission blocked, exhibit an elongated mitochondrial network along with reduced respiratorychain complexes, ATP depletion, and worsened heart failure. A similar phenotype appears in a Parkin-null Drosophila cardiac tube: defective mitophagy causes swollen mitochondria and culminates in dilated cardiomyopathy (Bhandari et al., 2014). Conversely, cardiac adenoviral overexpression of PINK1, which boosts mitophagy, attenuates pressure-overload-induced hypertrophy and fibrosis (Song et al., 2015), reinforcing the cardioprotective role of mitophagy. In the transverse aortic constriction (TAC) model, hemodynamic stress not only imposes mechanical tension but also activates neuro-humoral and paracrine cues that injure organelles such as the ER and mitochondria. TAC rapidly triggers LC3-dependent macroautophagy, which returns to baseline within 24 h (Shirakabe et al., 2016); mitophagy peaks 3 to 7 days after surgery and then wanes, temporally separated from the onset of ventricular failure (Nah et al., 2022). Post-operative administration of TAT Beclin 1 partially restores mitophagy and improves cardiac performance under pressure overload, indicating a protective window for mitophagy (Shoji-Kawata et al., 2013). Although Parkin-deficient mice develop more severe hypertrophy and systolic dysfunction than wild-type controls after TAC, this difference has been attributed to amplified endoplasmic-reticulum stress rather than impaired mitophagy (Soubannier et al., 2012), highlighting the multifaceted functions of Parkin. Chronic myocardial infarction causes volume overload, driving hypertrophy, chamber dilation, and failure. After myocardial infarction (MI), cardiac mitophagy depends on Parkin; Parkin loss curtails autophagic clearance of damaged mitochondria and thereby aggravates remodeling and functional decline (Kubli et al., 2013). PINK1 appears dispensable, because its absence does not hinder Parkin recruitment to injured mitochondria (Kubli et al., 2015). RAB9-mediated alternative mitophagy is engaged during pressure overload (Nah et al., 2022). ULK1 is a key regulator of this pathway, and cardiomyocyte-specific Ulk1 deletion precipitates earlier dysfunction after TAC (Nah et al., 2022). Mitophagy also requires intra-lysosomal DNase II to degrade mtDNA at acidic pH; reduced DNase II expression causes mtDNA accumulation that engages TLR9, promotes inflammation, and worsens pressure-overload heart failure, whereas TLR9 deficiency preserves function under the same stress (Melentijevic et al., 2017).

During the progression of cardiac hypertrophy and heart failure, mitophagy exhibits dynamic alterations that can be either protective or detrimental, depending on the context and regulatory balance. In the early phase, moderate activation of PINK1/Parkin-mediated mitophagy facilitates the clearance of damaged mitochondria, alleviates cellular stress, and delays ventricular remodeling. Conversely, mitophagy deficiency is frequently associated with ATP depletion, mitochondrial dysfunction, and structural myocardial damage. However, some studies suggest that the exacerbation of cardiac dysfunction in Parkin-deficient models may be more attributable to enhanced endoplasmic reticulum stress, highlighting the multifaceted roles of Parkin beyond mitophagy. In pressure overload models, mitophagy activation and resolution follow a distinct temporal pattern that is not synchronized with the onset of heart failure phenotypes, suggesting the existence of a therapeutic window for sustaining autophagic activity. Moreover, RAB9-mediated alternative mitophagy and DNase II–dependent degradation of mitochondrial DNA has been shown to alleviate myocardial injury, indicating additional layers of regulation. Collectively, the role of mitophagy in hypertrophy and heart failure is influenced by the magnitude, timing, and interplay of signaling pathways. Further research is needed to delineate its stage-specific functions and mechanistic interactions, which will be critical for refining targeted therapeutic strategies.

### Age related decline of cardiac mitophagy

During cardiac aging, the capacity for mitochondrial quality control declines, accompanied by tissue remodeling, reduced contractility, and diastolic dysfunction (Abdellatif et al., 2023). In hearts from 2-year-old mice, basal autophagosome formation is markedly diminished and mitochondria show structural damage (Liang et al., 2020). Likewise, in D-galactose-induced accelerated aging, both general autophagy and mitophagy are significantly lower, paralleling ventricular remodeling and impaired relaxation (Wang et al., 2023). Comprehensive genetic and pharmacological studies indicate that declining autophagy and mitophagy allow the buildup of dysfunctional mitochondria, thereby accelerating cardiac aging (Wang et al., 2021). Furthermore, cardiomyocyte specific ablation of the essential autophagy component ATG5 abolishes autophagosome formation and precipitates fatal cardiomyopathy accompanied by mitochondrial impairment and sarcomere disorganization (Taneike et al., 2010). Even a ~70% reduction of ATG5 in cardiomyocytes is sufficient to produce age-dependent cardiac decline and shortened lifespan, showing that insufficient autophagy alone can drive cardiac aging (Wang et al., 2021). Conversely, systemic overexpression of ATG5 elevates basal autophagy and lengthens lifespan in mice (Pyo et al., 2013) ; however, its specific effects on mitochondrial integrity within the heart remain unclear. Strengthening Parkin mediated mitophagy in the mouse myocardium enhances mitochondrial quality and postpones age related decline (Hoshino et al., 2013), whereas deficiency of PINK1, Parkin, BNIP3, or NIX results in excessive mitochondrial accumulation and contractile impairment (Kubli, Quinsay & Gustafsson, 2013). Although mitochondrial DNA lesions accumulate with advancing age, Parkin driven mitophagy makes only a modest contribution to mitochondrial genome surveillance (Filograna et al., 2024). In prematurely aged POLG^D275A^ mice, deletion of this pathway does not intensify mitochondrial defects or early aging traits in brain, skeletal muscle, or heart (Woodall et al., 2019). The basis of the age-related decline in mitophagy is multifaceted and study outcomes are sometimes inconsistent. Some investigations report lower Parkin abundance in aged murine hearts (Gao et al., 2021; Wang et al., 2018b), whereas others observe higher Parkin levels yet fewer autophagosomes (Liang et al., 2020). Parkin also ubiquitinates many proteins unrelated to mitophagy, indirectly shaping mitochondrial function (Peng et al., 2023). Down regulation of the essential autophagy factor ATG9B in aged hearts may further restrict autophagosome formation (Liang et al., 2020). Moreover, expression of the age regulated transcription factor MondoA declines with age and correlates with diminished autophagy and accelerated senescence; MondoA supports autophagy by repressing Rubcn, which encodes the inhibitory protein Rubicon (Yamamoto-Imoto et al., 2022). Collectively, these observations suggest that the progressive loss of autophagy and mitophagy during aging stems from several interacting mechanisms whose relative impact varies among tissues and comorbid conditions.

Taken together, cardiac aging is accompanied by a progressive decline in mitophagic capacity, resulting in the accumulation of mitochondrial damage, reduced systolic performance, and diastolic dysfunction. Loss or downregulation of core autophagy factors such as ATG5 precipitates severe cardiomyopathy, whereas enhancing Parkin-mediated mitophagy helps preserve mitochondrial quality and delay senescence. Nevertheless, findings on age-related changes in Parkin expression are inconsistent, and its direct contribution to mitochondrial DNA homeostasis appears limited. In addition, downregulation of aging-associated factors such as ATG9B and MondoA may indirectly suppress autophagic activity. Overall, the age-dependent erosion of mitophagy reflects perturbations across multiple molecular layers and signaling pathways; its relative contribution across tissues and in comorbid states warrants further clarification to define actionable therapeutic targets.

### Mitophagy in obesity linked cardiomyopathy and high fat diet

Obesity is a common metabolic disorder that frequently leads to diastolic dysfunction, myocardial hypertrophy and inflammation (Tan et al., 2020). When mitochondrial performance deteriorates, fatty acid uptake and oxidation become unbalanced, driving lipid droplet accumulation and lipotoxicity (D’Souza, Nzirorera & Kienesberger, 2016). Increasing evidence indicates that mitophagy is crucial for preserving mitochondrial quality in obesity-induced cardiomyopathy (Zhi et al., 2023). Cardiomyocyte specific autophagy deficient mice fed the same high fat diet (HFD) as wild type controls display more severe mitochondrial injury, including loss of membrane potential and reduced respiration, and a more rapid decline in cardiac performance (Tong et al., 2019). Parkin knockout mice maintained on a HFD display more severe cardiac hypertrophy and systolic dysfunction (Tong et al., 2019), underscoring the necessity of Parkin mediated mitophagy for adapting to lipid excess. Moreover, continuous HFD intake for 12 or 24 weeks markedly reduces myocardial Parkin abundance relative to a low fat regimen (Shao et al., 2020) and weakens the capacity to initiate mitophagy after ischemia followed by reperfusion (Thomas et al., 2019). Although cardiac autophagy and mitophagy increase in the early phase of HFD exposure, their activity declines after approximately 2 months (Eshima et al., 2017). The pathway is not irreversibly silenced; a cell penetrating Beclin 1 based peptide, TB 1, can reactivate mitophagy and mitigate the adverse cardiac effects of HFD (Tong et al., 2019). Members of the TFEB family, particularly TFE3, increase after 12 weeks of HFD in parallel with activation of alternative mitophagy (Tong et al., 2021). HFD augments nuclear TFE3 in cardiomyocytes, enabling it to bind the promoter of Rab9 (Tong et al., 2021). Cardiomyocyte specific deletion of TFE3 blocks this alternative route, confirming its dependence on TFE3 driven Rab9 transcription (Tong et al., 2021). During prolonged HFD exposure, RAB9 dependent alternative mitophagy helps maintain mitochondrial quality and affords partial protection in murine obesity related cardiomyopathy (Nahapetyan et al., 2019). Chronic HFD also elevates Drp1 phosphorylation at Ser616 and stimulates alternative mitophagy at mitochondria associated membranes (Tong et al., 2023); the same modification is observed in hearts from obese patients (BMI greater than 30 kg/m^2^) (Tong et al., 2023), suggesting that Drp1 is a potential target for modulating autophagy in long term obesity related heart disease. High fat feeding is not universally beneficial. In mice lacking frataxin specifically in the heart, an HFD fails to protect against cardiomyopathy (Billia et al., 2011). Moreover, sustained lipid surplus worsens the functional impairment produced by pressure overload (Li et al., 2022a).

Collectively, in cardiomyopathy associated with a high-fat diet or obesity, mitophagy provides protection by preserving mitochondrial function, countering lipotoxicity, and alleviating myocardial metabolic derangements. Loss or downregulation of Parkin exacerbates ventricular hypertrophy and dysfunction induced by a high-fat diet, whereas peptide activators of Beclin 1 and the RAB9 alternative autophagy pathway mediated by TFE3 can restore autophagic activity and improve cardiac performance. Phosphorylation of Drp1 represents a pivotal regulatory node for alternative autophagy and shows a similar pattern in hearts from obese patients, which highlights translational potential. However, any benefit observed under a high-fat diet is context dependent; in certain genetic backgrounds such as frataxin deficiency the effect may be abolished, and lipid overload acting together with other stresses can further impair cardiac function. Overall, the dynamic regulation of mitophagy in lipid-rich settings calls for a systematic appraisal that integrates metabolic status, disease stage, and specific genetic context to define actionable intervention points.

### Sex differences in cardiac mitophagy

Recent human and animal studies indicate sex differences in cardiac mitochondrial phenotype and energetics, which relate to diastolic function and provide a biological basis for sex specific regulation of mitophagy (Cao et al., 2022). In the setting of ischemia–reperfusion, activation of the membrane receptor GPER1 attenuates mitochondrial injury, limits stress induced mitophagy including modulation of the PINK1 and Parkin axis, reduces infarct size, and improves cardiac function, indicating that sex hormones can tune the intensity and threshold of autophagy within the myocardium (Feng et al., 2017). Endothelial models further show that estrogen acting through ERα enhances RAB9 dependent alternative mitophagy in the perilysozomal compartment, which mitigates inflammatory burden and vascular wall stress, suggesting that distinct cardiovascular cell types deploy different routes for sex related mitochondrial clearance (Sasaki et al., 2021). Consistent findings in experimental pulmonary arterial hypertension reveal sex differences in Parkin abundance and mitophagy markers that associate with right ventricular remodeling and functional adaptation, thereby linking sex to mitophagy in pressure loaded heart disease (Chavan & Muth, 2021). The heart is a high oxygen consuming organ with measurable basal mitophagy at steady state, and parts of this process proceed in the absence of PINK1, which provides a framework for interpreting differential effects of sex hormones on clearance pathways under resting and stress conditions (McWilliams et al., 2018). Taken together, these observations support the working hypothesis that in estrogen dominant settings, tempering excessive PINK1 or Parkin driven mitophagy may help avert energetic depletion and limit injury, whereas in estrogen deficiency or male settings, cautiously enhancing mitophagy flux may better support post injury repair, although these predictions require direct quantification in cardiomyocytes (Cao et al., 2022; Feng et al., 2017). Given the presence of PINK1 independent basal mitophagy in the myocardium, evaluation of sex differences should rely on *in vivo* flux tracing and multi parameter quantitative frameworks to avoid biased conclusions from single molecular readouts (McWilliams et al., 2018). Overall, cross model evidence indicates that sex hormones and their receptors shape the cardiovascular mitophagy network by tuning the PINK1 and Parkin axis or engaging the RAB9 dependent alternative pathway, which provides a mechanistic foundation for sex stratified cardioprotective strategies and therapeutic interventions.

### Cardiac mitophagy in diabetes

Cardiac mitophagy is broadly dysregulated in diabetes, presenting as either suppressed flux or compensatory activation, and both patterns are linked to disturbed energy metabolism and structural remodeling (Li et al., 2024). In mouse models of type 2 diabetes, administration of a hydrogen sulfide donor induces persulfidation of the deubiquitinase USP8, stabilizes Parkin, and restores autophagic flux through the PINK1 and Parkin axis, thereby attenuating myocardial fibrosis and diastolic dysfunction (Sun et al., 2020). In streptozotocin models and high glucose exposed cardiomyocytes, upregulation of the stress sensor Sestrin2 promotes Parkin dependent mitophagy, lowers mitochondrial oxidative stress and apoptosis, and improves cardiac functional readouts (Zhuang et al., 2025). In diabetes related myocardial injury, melatonin enhances mitophagy and mitochondrial homeostasis, reduces reactive oxygen species accumulation and histological damage, and supports the therapeutic potential of targeting autophagy (Wang et al., 2018c). Conversely, cardiomyocyte loss of Sirtuin 3 suppresses Parkin mediated mitophagy and aggravates mitochondrial fragmentation and dysfunction in diabetic cardiomyopathy, underscoring the role of deacetylation pathways in maintaining autophagic balance (Guan et al., 2024). In tested type 2 diabetes models, a ketogenic diet augments autophagy and mitochondrial clearance, reduces tumor necrosis factor related inflammation, and improves left ventricular systolic function, indicating that metabolic interventions can indirectly correct autophagic defects (Mu et al., 2020). Receptor mediated mechanisms also contribute to mitochondrial quality control in diabetic hearts, as fucoxanthin activates BNIP3 and NIX to drive selective autophagy and thereby mitigates fibrosis and diastolic impairment (Zheng et al., 2022a). Under prolonged diabetes, uridine therapy upregulates Pink1 expression, improves mitochondrial turnover and oxidative phosphorylation efficiency, and supports strategies that enhance autophagy related signaling to restore energy supply (Belosludtseva et al., 2022). Taken together, stratified modulation of the PINK1 and Parkin axis and receptor pathways such as BNIP3 and NIX, combined with metabolic regulators including Sirtuin 3, may enable targeted correction of mitophagic flux in the diabetic myocardium and translate into functional benefit.

### Mitophagy in coronary artery disease and atherosclerosis

Mitochondrial quality control failure in vascular wall cells is a central determinant of the initiation and progression of coronary artery disease and atherosclerosis, where mitophagy can operate as an adaptive defense yet may amplify injury when stress becomes excessive (Coon et al., 2022). On the endothelial side, prolonged laminar shear stress preserves an anti-inflammatory phenotype and barrier function by sustaining mitochondrial homeostasis and programs linked to selective autophagy, indicating that basal mitophagy helps restrain formation of a proatherogenic milieu (Coon et al., 2022). Hyperglycemic stress induces PINK1 and Parkin dependent excessive mitophagy in human endothelial cells *in vitro* and coincides with endothelial dysfunction, whereas the glucagon like peptide one receptor agonist liraglutide suppresses this pathway and improves endothelial functional readouts, suggesting that moderate down tuning of excessive flux may favor vascular homeostasis under metabolic stress (Zhang et al., 2022b). Synthesis of current evidence further indicates that infection related signals drive mitochondrial stress and modulate endothelial inflammation and barrier responses through autophagy and mitophagy, providing a mechanistic bridge between the inflammatory axis of atherogenesis and mitochondrial quality control (Zheng et al., 2025). On the macrophage side, stimulation by gut derived bacteria enhances proinflammatory polarization and activates the NLRP3 inflammasome together with mitochondrial dysfunction and impaired quality control, thereby aggravating atherosclerotic burden and underscoring the importance of sufficient mitophagy to restrain sterile inflammation (Duan et al., 2022). Taken together, findings from endothelial and macrophage models support the concept that different arterial wall cell types have distinct optimal set points for mitophagic flux, and that both insufficiency and excess can magnify oxidative stress and sterile inflammation to drive plaque formation and progression (Coon et al., 2022; Duan et al., 2022). From an interventional perspective, preserving laminar flow related basal mitochondrial homeostasis and selective mitophagy may serve as a biological reference for endothelial targeting, while curbing abnormally heightened PINK1 and Parkin signaling under hyperglycemia or metabolic syndrome may improve nitric oxide signaling and endothelial function (Coon et al., 2022; Zhang et al., 2022b). In parallel, coordinated control of mitochondrial stress and inflammasome activation in macrophages requires promoting appropriate mitophagy while maintaining pathogen clearance capacity to limit inflammatory amplification driven by mitochondrial reactive oxygen species and mitochondrial DNA (Duan et al., 2022; Zheng et al., 2025). Overall, convergent studies delineate a basal, moderate, and context dependent mitophagic flux as a determinant of vascular homeostasis and provide actionable directions for context specific and cell type specific modulation of mitophagy in coronary artery disease and atherosclerosis.

## Therapeutic targeting of cardiac mitophagy

Robust pre-clinical evidence indicates that pharmacological approaches designed to enhance autophagy, particularly mitophagy, can improve mitochondrial performance, sustain myocardial contractility and extend lifespan (Eisenberg et al., 2016; Zhang et al., 2022a). The most recently studied compounds aimed at restoring mitochondrial quality control in diseased hearts are summarized in Table 1.

**Table 1:** Pharmacological modulators of cardiac mitophagy: experimental models and mechanistic effects.

Drug	Model	Effect	References
Melatonin	Rat myocardial ischemia reperfusion model	Suppresses excessive PINK1-Parkin dependent mitophagy *via* the Apelin/SIRT3 pathway, lowering oxidative stress and limiting infarct size.	Wang et al. (2024)
Metformin	Mouse chronic intermittent hypoxia induced myocardial injury	AMPKα2 activation leads to HIF-1α phosphorylation and reduced nuclear localization, thereby inhibiting IH induced cardiac mitophagy and improving contractility.	Moulin et al. (2025)
MitoQ	Rat myocardial infarction induced chronic heart failure	Mitochondria targeted antioxidant; together with exercise normalizes MFN2, PINK1 and FIS1, rebalance fusion/fission, and promotes moderate mitophagy, improving cardiac performance.	Rostamzadeh et al. (2024)
Rapamycin	Rat chronic heart failure model	Inhibits mTORC1 and ER-stress pathways, promoting global autophagy and attenuating apoptosis; mitophagy was not specifically assessed in this study	Gao et al. (2020)
Spermidine	Aged mice and Dahl salt sensitive rats	Chronic dietary intake enhances cardiac autophagy/mitophagy and mitochondrial respiration, reduces hypertrophy, preserves diastolic function and delays cardiac aging	Eisenberg et al. (2016)
Urolithin A	Diet-induced obese mice (metabolic cardiomyopathy)	Activates PINK1/Parkin-dependent mitophagy, clears damaged mitochondria, reverses diastolic dysfunction and limits remodeling	Huang et al. (2023)

Accumulating experimental work indicates that precisely modulated cardiac mitophagy confers protection in diverse injury settings. In a rat model of acute myocardial ischemia followed by reperfusion, melatonin given during the reperfusion phase activated Apelin/SIRT3 signaling and was associated with suppression of marker-defined activity within the PINK1 and Parkin axis, reducing infarct size and improving ventricular function (Wang et al., 2024). In C57BL/6 mice exposed to chronic intermittent hypoxia, metformin limited increases in mitophagy-associated markers *via* AMPKα2-mediated phosphorylation of HIF-1α, thereby maintaining systolic performance (Moulin et al., 2025). When isoproterenol provoked acute myocardial injury in rats, the mitochondria-targeted antioxidant MitoQ combined with moderate endurance training, rebalanced fusion and fission proteins and increased mitophagy-associated readouts, leading to better wall motion and ejection fraction (Rostamzadeh et al., 2024). Rapamycin therapy in rats with chronic heart failure suppressed mTORC1 activity, restored the balance between autophagy and apoptosis, and lowered cardiomyocyte death, although mitophagy itself was not specifically quantified in that study (Gao et al., 2020). In aged or pressure-overloaded mice, sustained spermidine supplementation triggered BNIP3/FUNDC1-dependent mitophagy, enhanced mitochondrial respiratory efficiency and delayed cardiac aging (Eisenberg et al., 2016). Lastly, in a high-fat-diet model of obesity-related cardiomyopathy, punicic acid A activated the PINK1/Parkin pathway to accelerate removal of damaged mitochondria, thereby correcting diastolic dysfunction and metabolic abnormalities (Huang et al., 2023). Several of these agents are already used clinically or available as nutraceuticals, underscoring translational potential, yet most studies inferred effects on mitophagy from static protein abundance, colocalization, or ultrastructural changes rather than validated *in vivo* flux reporters. Future preclinical and early-phase clinical work should incorporate standardized flux assays, including phospho-ubiquitin measurements under lysosomal blockade, genetically encoded reporters, or live tracking of mitochondria–lysosome contacts, to distinguish pathway engagement from *bona fide* changes in mitophagy flux. Taken together, these studies demonstrate that context-specific restoration of mitophagic flux can recalibrate myocardial energy metabolism and lessen structural injury at the molecular level, positioning mitochondrial quality control as an emerging pharmacological target in cardiovascular disease.

## Conclusions and future perspectives

Mitophagy is a critical defense that preserves energy supply and structural integrity in cardiomyocytes. When mitochondrial injury surpasses basal clearance capacity, the PINK1/Parkin dependent phosphorylated ubiquitin amplification loop, together with receptor guided BNIP3, NIX and FUNDC1 routes, recognizes, encloses and delivers damaged mitochondria to lysosomes, thereby restoring intracellular equilibrium. Additional mechanisms, which include complexes formed by Beclin 1, PI3K and ULK1, externalized cardiolipin and lipid droplet related signaling networks, further guide autophagosome targeting and formation, ensuring efficient and selective disposal of injured organelles. An imbalance of mitophagy amplifies cardiac remodeling through oxidative stress, inflammatory cascades and programmed cell death, and exerts a double-edged influence in hypertrophy, heart failure, cardiac aging and diet induced metabolic cardiomyopathy. During acute ischemia reperfusion, a brief surge in mitophagy rapidly removes depolarized mitochondria that generate ROS and accumulate calcium, suppressing NLRP3 inflammasome assembly and limiting cardiomyocyte loss. Persistent activation, however, depletes ATP and initiates necrotic as well as apoptotic pathways. Chronic pressure overload, aging and obesity share a trajectory in which early compensatory autophagy later collapses. Alternative routes such as Rab9/ULK1 signaling and phosphorylation of Drp1 at Ser616 provide limited backup when canonical flux is obstructed, yet cannot prevent progressive ventricular remodeling. Studies of small molecules and natural products such as melatonin, metformin, MitoQ, rapamycin, spermidine and urolithin A show that precise modulation of selected steps within the autophagic pathway can increase ATP generation, reduce fibrosis and slow the decline of contractile performance in experimental models. Future therapeutic strategies therefore need to define an optimal dosing window that balances stimulation and suppression of mitophagy, taking into account the patient’s metabolic phenotype and stage of disease.

These converging lines of evidence indicate that stage-specific modulation of myocardial mitophagy has clear translational potential. In acute ischemia–reperfusion, brief enhancement of mitophagy promotes removal of depolarized mitochondria and attenuates oxidative stress and inflammation, supporting its use as an adjunct to reperfusion therapy. In chronic pressure overload and cardiac aging, an early, transient rise in autophagy is followed by collapse, suggesting that sustaining a moderate flux during early remodeling may slow functional decline. In metabolic cardiomyopathy, lipotoxicity and impaired mitophagy reinforce one another; activating the PINK1/Parkin pathway or engaging the RAB9-dependent alternative route may improve diastolic function and correct metabolic derangement. A practical roadmap should stratify patients by metabolic phenotype and disease stage, combine mitophagy modulation with standard heart-failure care, apply short-course interventions during reperfusion, and consider longer-term low-dose regimens in conditions dominated by disordered lipid metabolism such as obesity or heart failure with preserved ejection fraction. Initial candidates with favorable safety profiles and accessibility include metformin, melatonin, rapamycin, and spermidine, with systematic evaluation of synergy with structured exercise. Successful implementation requires actionable monitoring. In the near term, circulating mitochondrial DNA, phosphorylated ubiquitin, myocardial BNIP3 or FUNDC1 expression, and cardiac T1 and T2 mapping can serve as surrogate readouts, while development of molecular imaging probes that dynamically report autophagic flux should be a longer-term goal. Because excessive autophagy may precipitate energy depletion and cell death, study designs should prioritize careful control of dose, timing, and cardiac-targeted delivery. Mechanism-focused pilot trials in enriched high-risk populations, using composite endpoints linked to energy metabolism and fibrosis, can de-risk translation. An intervention framework built around timing, stratification, dose, and monitoring offers a path to convert molecular insights on mitophagy into actionable cardiovascular therapies.

## Supplemental Information

10.7717/peerj.20700/supp-1 Supplemental Information 1 Mitophagy regulation in pressure overload, cardiac aging and obesity-related cardiomyopathy. A. Loss of Parkin impairs mitophagy, causing mitochondrial swelling and the development of DCM. Cardiac overexpression of PINK1 enhances mitophagy and attenuates pressure overload–induced cardiac hypertrophy and fibrosis. The cell-penetrating peptide TAT–Beclin 1 restores mitophagy and improves cardiac function under pressure overload. B. In the aging heart, mitophagy is markedly suppressed, leading to the accumulation of damaged mitochondria and thereby accelerating cardiac aging. Cardiomyocyte-specific deletion of the core autophagy proteins ATG5, PINK1, Parkin or BNIP3/NIX results in defective mitophagy accompanied by mitochondrial dysfunction. In aged myocardium, reduced expression of the autophagy-related protein ATG9B and the age-associated transcription factor MondoA correlates with diminished autophagic activity and an aggravated premature aging phenotype. C. HFD feeding markedly decreases cardiac Parkin levels, blunts the capacity to activate mitophagy and promotes obesity-related cardiomyopathy. In response to HFD, TFE3 is upregulated and binds to the Rab9 promoter to drive Rab9 transcription, thereby engaging RAB9-dependent alternative mitophagy and conferring protection in obese cardiomyopathy models. HFD also enhances Drp1 phosphorylation at Ser616 and promotes alternative mitophagy at MAMs. DCM, dilated cardiomyopathy; HFD, high-fat diet; TAT–Beclin 1, TAT-fused Beclin 1–derived peptide; ATG5, autophagy related 5; ATG9B, autophagy related 9B; BNIP3, BCL2/adenovirus E1B 19 kDa-interacting protein 3; NIX, NIP3-like protein X (BNIP3L); TFE3, transcription factor E3; Rab9, Ras-related protein Rab-9; RAB9, Rab9 GTPase; Drp1, dynamin-related protein 1; MAM, mitochondria-associated ER membrane.

## Abbreviations

AMPK: AMP-activated protein kinase
ATG5: autophagy-related protein 5
BNIP3: BCL2/adenovirus E1B 19 kDa-interacting protein 3
CL: cardiolipin
FUNDC1: FUN14 domain containing 1
HFD: high-fat diet
HIF-1α: hypoxia-inducible factor 1 alpha
I/R: ischemia/reperfusion
LC3: microtubule-associated protein 1A/1B-light chain 3
LD: lipid droplet
mtROS: mitochondrial reactive oxygen species
NIX: NIP3-like protein X
NLRP3: NOD-like receptor family pyrin domain containing 3
Nrf2: nuclear factor erythroid 2-related factor 2
PGC-1α: peroxisome proliferator-activated receptor gamma coactivator 1-alpha
PI3K: phosphatidylinositol 3-kinase
PINK1: PTEN-induced kinase 1
Parkin: E3 ubiquitin-protein ligase Parkin
RAB9: Ras-related protein Rab-9
RIPK3: receptor-interacting serine/threonine-protein kinase 3
ROS: reactive oxygen species
SIRT3: sirtuin 3
SIRT6: sirtuin 6
ULK1: Unc-51-like autophagy activating kinase 1
USP30: ubiquitin-specific protease 30

## Additional Information and Declarations

### Data Availability

The following information was supplied regarding data availability:

This is a literature review.
