# Supplementary figures and images for: Mastering cardiomyocyte mitophagy: molecular governance, pathological derailment and therapeutics
Source: PeerJ. 2026 Feb 10;14:e20700. doi: 10.7717/peerj.20700 (PMC12903907; doi:10.7717/peerj.20700)

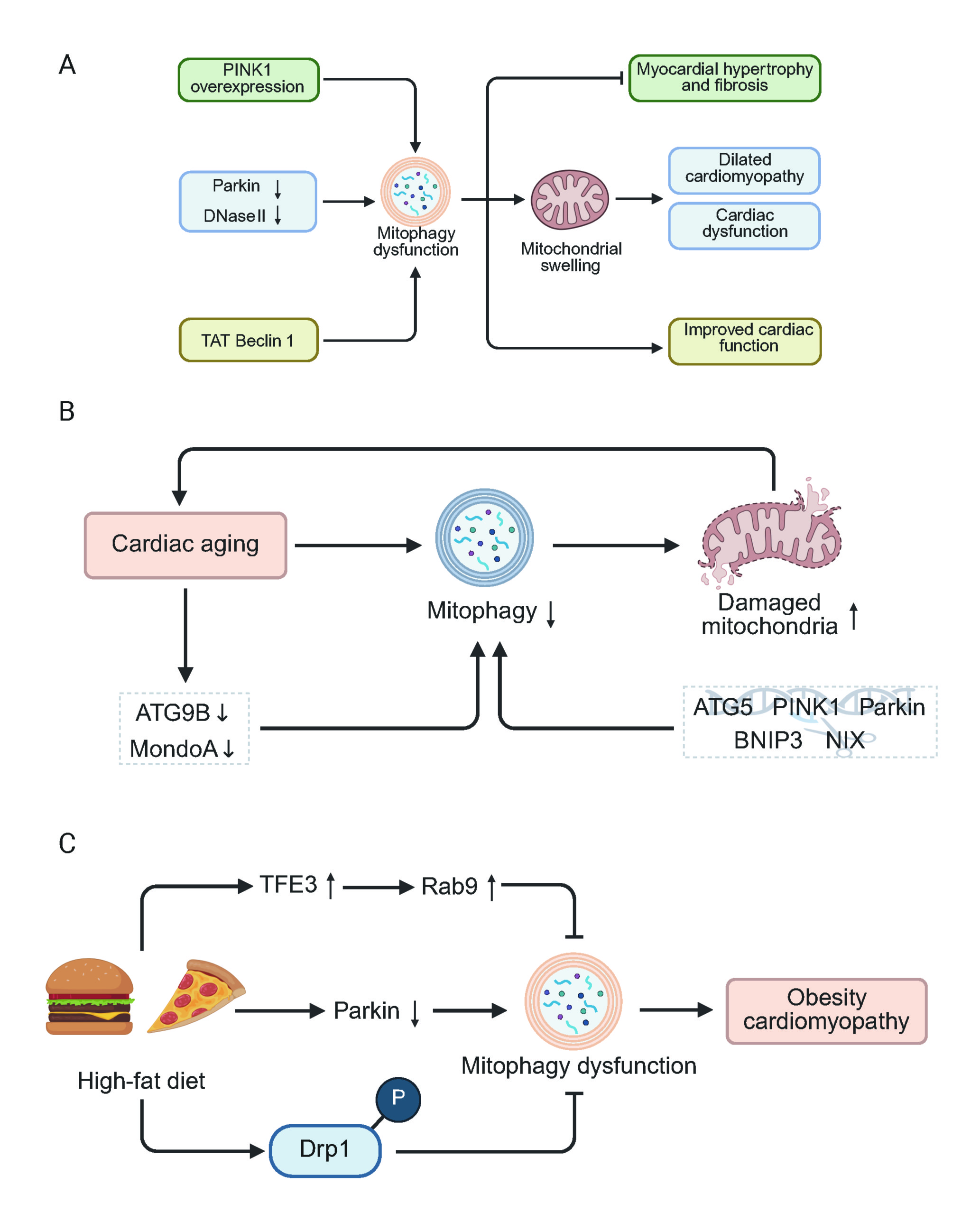

Supplement: Supplemental Information 1 — A. Loss of Parkin impairs mitophagy, causing mitochondrial swelling and the development of DCM. Cardiac overexpression of PINK1 enhances mitophagy and attenuates pressure overload–induced cardiac hypertrophy and fibrosis. The cell-penetrating peptide TAT–Beclin 1 restores mitophagy and improves cardiac function under pressure overload. B. In the aging heart, mitophagy is markedly suppressed, leading to the accumulation of damaged mitochondria and thereby accelerating cardiac aging. Cardiomyocyte-specific deletion of the core autophagy proteins ATG5, PINK1, Parkin or BNIP3/NIX results in defective mitophagy accompanied by mitochondrial dysfunction. In aged myocardium, reduced expression of the autophagy-related protein ATG9B and the age-associated transcription factor MondoA correlates with diminished autophagic activity and an aggravated premature aging phenotype. C. HFD feeding markedly decreases cardiac Parkin levels, blunts the capacity to activate mitophagy and promotes obesity-related cardiomyopathy. In response to HFD, TFE3 is upregulated and binds to the Rab9 promoter to drive Rab9 transcription, thereby engaging RAB9-dependent alternative mitophagy and conferring protection in obese cardiomyopathy models. HFD also enhances Drp1 phosphorylation at Ser616 and promotes alternative mitophagy at MAMs. DCM, dilated cardiomyopathy; HFD, high-fat diet; TAT–Beclin 1, TAT-fused Beclin 1–derived peptide; ATG5, autophagy related 5; ATG9B, autophagy related 9B; BNIP3, BCL2/adenovirus E1B 19 kDa-interacting protein 3; NIX, NIP3-like protein X (BNIP3L); TFE3, transcription factor E3; Rab9, Ras-related protein Rab-9; RAB9, Rab9 GTPase; Drp1, dynamin-related protein 1; MAM, mitochondria-associated ER membrane. [file peerj-14-20700-s001.jpg]
